# Shifting the Gears of Metabolic Plasticity to Drive Cell State Transitions in Cancer

**DOI:** 10.3390/cancers13061316

**Published:** 2021-03-15

**Authors:** Zhengwei Wu, Yi Fei Lee, Xun Hui Yeo, Ser Yue Loo, Wai Leong Tam

**Affiliations:** 1 Cancer Science Institute of Singapore, National University of Singapore, 14 Medical Drive, Singapore 117599, Singapore; zwwu@gis.a-star.edu.sg (Z.W.); yeoxh@gis.a-star.edu.sg (X.H.Y.); 2 Genome Institute of Singapore, 60 Biopolis Street, Singapore 138672, Singapore; lee_yi_fei_from.tp@gis.a-star.edu.sg; 3 School of Biological Sciences, Nanyang Technological University, 60 Nanyang Drive, Singapore 637551, Singapore; 4 Department of Biochemistry, Yong Loo Lin School of Medicine, National University of Singapore, 8 Medical Drive, Singapore 117597, Singapore

**Keywords:** cellular plasticity, phenotype switching, cancer stem cells, epithelial–mesenchymal transition, cell state transition, therapy resistance

## Abstract

**Simple Summary:**

Metabolic adaptation by cancer cells is enabled through the rewiring of metabolic processes, thereby allowing them to survive and thrive in diverse tissue microenvironments. It is also exploited to maintain cancer stemness, drive epithelial–mesenchymal transition, and gain therapy resistance. These critical cellular events are pertinent to the various steps of cancer progression. Mechanistic insights into nutrient addiction arising from such metabolic rewiring have revealed therapeutic vulnerabilities that can be exploited as novel treatment modalities or for drug development. This review discusses concepts and principles of metabolic plasticity and highlights current preclinical and clinical strategies aimed at targeting these metabolic derangements.

**Abstract:**

Cancer metabolism is a hallmark of cancer. Metabolic plasticity defines the ability of cancer cells to reprogram a plethora of metabolic pathways to meet unique energetic needs during the various steps of disease progression. Cell state transitions are phenotypic adaptations which confer distinct advantages that help cancer cells overcome progression hurdles, that include tumor initiation, expansive growth, resistance to therapy, metastasis, colonization, and relapse. It is increasingly appreciated that cancer cells need to appropriately reprogram their cellular metabolism in a timely manner to support the changes associated with new phenotypic cell states. We discuss metabolic alterations that may be adopted by cancer cells in relation to the maintenance of cancer stemness, activation of the epithelial–mesenchymal transition program for facilitating metastasis, and the acquisition of drug resistance. While such metabolic plasticity is harnessed by cancer cells for survival, their dependence and addiction towards certain metabolic pathways also present therapeutic opportunities that may be exploited.

## 1. Introduction

Cellular plasticity defines the ability of cells to adopt different characteristics along a phenotypic spectrum [1]. An underlying process in embryonic development, tissue regeneration and wound healing, it is increasingly apparent that cellular plasticity is exploited and exhibited by tumor cells to gain growth, competitive, and adaptive advantages [2]. These include adapting to different nutrient availabilities in the tumor microenvironment, escaping immune surveillance, switching cell states during epithelial–mesenchymal transition (EMT), invading a secondary site, and gaining cancer drug resistance [3,4,5]. Cellular plasticity can be induced by genetic and epigenetic alterations, external environmental stimuli, or in response to drug treatment [1]. These changes culminate in tumor heterogeneity, metastasis, and therapeutic resistance, thus rendering treatment increasingly difficult [5]. Developing strategies to control or deter cellular plasticity may be essential for achieving better treatment outcomes.

More recently, altered metabolic requirements have been implicated in a spectrum of cell states [6]. The adaptive nature of heterogenous cancer cell populations during disease progression necessitates shifts in metabolic phenotypes. In fact, cancer metabolism, which is the study of metabolic alterations in tumor cells, has been enshrined as a hallmark of cancer [7,8]. The metabolic reprogramming of tumor cells and their crosstalk with the surrounding microenvironment is now recognized as a determinant for tumors to initiate, grow, adapt, and metastasize [9]. Otto Warburg first reported altered metabolism during which tumor cells avidly uptake glucose and harness aerobic glycolysis for rapid proliferation [10,11]. Glucose transporters and glycolytic enzymes are upregulated to utilize glucose for the synthesis of nucleotides, amino acids, and lipids. Alternate carbon sources such as glutamine, branched-chain amino acids, fatty acids, and lactate have also been reported to fuel the growth of tumor cells, contribute towards cancer stemness or drive tumorigenesis [12]. Likewise, such metabolic reprogramming is seen in cell state transitions that contribute towards metastasis and confer drug resistance [13,14].

Here, we explore how cancer metabolism is altered during events of cellular plasticity, mainly focusing on metabolic adaptations that are exploited by: (i) cancer stem cells (CSCs); (ii) cell state changes during EMT; and (iii) drug resistant cancer cells. Insights into the roles that metabolic reprogramming plays in the adaptive nature of cancer cells may reveal new metabolic targets for drug development, or enable the redeployment of therapeutic options that can disrupt cancer cell metabolism in a specific and targeted manner.

## 2. Metabolic Plasticity Confers Adaptive Advantages to Cancer Stem Cells

Cancer stem cells (CSCs) are a subpopulation of cells within tumors that have the ability to self-renew, differentiate to other cell types, and are tumorigenic when transplanted to a new host at limiting cell dilution frequencies [15]. They possess distinct metabolic signatures, and are thought to contribute towards intra-tumoral heterogeneity, tumor relapse, and drug resistance [16,17,18,19,20,21]. While glycolysis and glutaminolysis are markedly elevated in bulk tumor cells to cater to increased ATP and NADPH demand [8,22], CSCs are phenotypically and functionally distinct. Hence, the manner by which they utilize nutrients for biosynthetic and energetic processes are expectedly quite different. Numerous reports suggest that CSCs are markedly more glycolytic, more reliant on oxidative phosphorylation (OxPhos), and have altered lipid and amino acid metabolisms, as compared to differentiated cancer cells [20].

Being more glycolytic in nature than bulk tumor cells, CSCs readily adapt to starvation or hypoxia and can outcompete non-CSCs under stress conditions [23]. The glucose-induced expression of specific genes relating to glucose metabolism (c-Myc, Glut-1, Hexokinase 1 (HK1), Hexokinase 2 (HK2) and PDK-1) could cause an increase in the CSC population [24]. This was shown to be driven by hypoxia-inducible factor I alpha (HIF1α), MYC and OCT4, which promoted the synthesis of glycolytic enzymes and proteins [25,26,27,28]. Under hypoxia, CSCs appeared to adopt an elevated glycolytic profile that was mediated by HIF1α and the Akt/mTOR/β-catenin stem cell pathway to gain a competitive edge over non-CSCs [29,30]. Therefore, glycolysis inhibition has been sought as a potential therapeutic option for overcoming cancer stemness.

Conversely, there is also growing evidence that CSCs may adopt a less glycolytic profile and preferentially rely on mitochondrial-powered oxidative phosphorylation (OxPhos) [14,31,32]. While they are in a quiescent state or subjected to adverse tumor microenvironmental conditions such as limited glucose availability, CSCs may adapt by inducing a metabolic switch via the activation of the transcription factor, peroxisome proliferator-activated receptor gamma coactivator 1-alpha (PGC1α), to rely more heavily on OxPhos for ATP production [33,34]. This is especially vital if CSCs are to persist both in primary tumors and also thrive at distant metastatic sites as metastasis-initiating cells [35]. Reconciling this paradox, more recently, CSCs were found to display stronger signatures of both glycolysis and OxPhos than non-CSCs [36]. In liver CSCs, glycolytic enzymes such as HK2, phosphofructokinase (PFK1) and pyruvate kinase (PKM), as well as OxPhos were all upregulated. This served to produce more pyruvate, which could be converted into acetyl-CoA to drive the TCA cycle for ATP production [36]. In CD133-expressing glioblastoma stem cells (GSCs), levels of an oncofetal protein–insulin-like growth factor 2 mRNA-binding protein (Imp2) were elevated [37]. Imp2 was responsible for OxPhos maintenance and regulated mitochondrial function through the post-transcriptional regulation of mitochondrial respiratory complexes [37]. Under hypoxia, when glycolysis was increased, the binding of Imp2 at target mRNAs (such as COX7b, COX16 and HMGA1) was enhanced to support OxPhos even under low oxygen tension [37].

The control of metabolic alterations may occur through epigenetic gene regulation pathways as well. For instance, in basal-like breast cancer, CSCs switched to rely more on glycolysis via the promoter methylation of fructose-1,6-biphosphatase (FBP1) by the Snail-G9a-Dnmt1 complex when OxPhos was inhibited [38]. The repression of FBP1 in these stem-like cells further increased stemness, tumorigenicity, epithelial-to-mesenchymal transition (EMT), and resistance to apoptosis [38,39,40]. The maintenance of a hybrid glycolysis and OxPhos phenotype provided the opportunity to harness both metabolic pathways for energy and biomass production. These studies provided an interesting glimpse into how CSCs could engage a hybrid glycolysis and OxPhos mode of energetic adaption to gain growth advantage over non-stem cells, as well as supporting metastasis [41,42].

The availability of nutrients in the tumor microenvironment is a critical determinant for maintaining stemness, driving tumorigenicity, and reshaping the metabolic signatures of CSCs. Other than glucose, CSCs display metabolic plasticity by adopting additional distinct metabolic pathways and utilizing specific metabolites for their biochemical needs. Glutamine availability was shown to maintain the stemness of “side population” cells in non-small cell lung cancer (NSCLC) and pancreatic cancer via redox homeostasis and the Wnt/β-catenin pathway [43]. By supplementation with L-asparaginase, which converts glutamine to glutamate, the tumor-initiating potential of the CSCs was severely crippled [43]. Alternate carbon sources, such as ketone bodies and lactate, were also shown to support CSC-like breast cancer cells [44,45]. Monocarboxylate transporter 1 (MCT-1) inhibition, which restricted uptake of ketone bodies and lactate, or treatment with mitoketoscins, which disrupted mitochondrial function, led to tumor shrinkage and a loss of stemness [45,46]. Along similar observations, the inhibition of the methionine cycle rate-limiting enzyme, methionine adenosyltransferase 2A (MAT2A), which led to a block in methionine utilization, was useful in preventing cancer relapse [20]. More interestingly, in relation to diet and the control of metabolic plasticity in CSCs, dietary and exogenously supplemented methionine could support tumor initiation and relapse in NSCLC, whereas a methionine-restricted diet, strikingly, reversed disease progression [20,47]. These studies shed light on the utilization of alternative fuels as a manifestation of metabolic adaptation in CSCs.

Beyond glucose and amino acids, metabolic reprogramming in CSCs also involves the selective utilization of lipids. Breast CSCs and leukemia-initiating cells appeared to possess enhanced fatty acid oxidation (FAO) as compared to non-CSCs for maintenance of stemness [48,49]. FAO refers to the sequential breakdown of fatty acids into acetyl-CoA units that feed into the TCA cycle. Lipophagy—the process of fusing lipid droplets with autophagosomes to release stored fatty acids—is an interesting new mechanism, which enables CSCs to rapidly react to metabolic stress through activating FAO [14]. Besides upregulating FAO, CSCs can exploit de novo lipogenesis and lipid droplet storage to meet their increased demand for lipids. Lipid droplets are endoplasmic reticulum-derived organelles that are beginning to gain prominence as a metabolic adaption for CSCs [50,51,52]. Higher levels of lipid droplets in colorectal, breast and ovarian CSCs were correlated with their increased tumorigenic potential [51,52,53]. Here, NF-κB signaling upregulated the expression of lipid desaturases stearoyl-CoA desaturase-1 (SCD1) and Δ6, which led to higher levels of unsaturated fatty acids and lipid droplets [53]. These lipid desaturases also contributed to the stemness in ovarian CSCs, as the pharmacological inhibition of desaturases resulted in the selective elimination of CSCs [53].

Cancer cells tend to produce high levels of reactive oxygen species (ROS) as a consequence of oncogenic transformation [54]. They may adapt to increased ROS levels by activating antioxidant pathways. As CSCs may deploy enhanced OxPhos, they likely require mechanisms to cope with increased oxidative stress induced by the elevated production of ROS beyond basal levels encountered by bulk cancer cells. Using a liver cancer mouse model in which tumor-initiating stem-like cells (TICs) were driven by NANOG expression, it was discovered that a high-cholesterol, high-fat Western diet resulted in transactivation of NANOG. This resulted in a metabolic switch involving the downregulation of OxPhos and the concomitant upregulation of fatty acid oxidation (FAO) [55]. NANOG promoted FAO by binding to the *Acadv1* mitochondrial gene locus; this contributed to the cell’s antioxidant defenses through the production of the reducing agent, NADPH [55]. Consequently, the silencing of NANOG reduced FAO, resulting in the loss of cancer stemness [55]. By maintaining low ROS levels, NANOG could be central in the antioxidant defense of CSCs to maintain their self-renewal property amidst a switch to the Western diet. Other than downregulating OxPhos, CSCs may upregulate the expression of antioxidant genes and increase the production of antioxidants in response to high ROS levels [56,57]. In gastrointestinal cancer stem-like cells, the ubiquitous stem cell marker, CD44, was demonstrated to increase cysteine uptake via interaction with a glutamate–cystine transporter (xCT); this drove the synthesis of antioxidant, glutathione (GSH), likely in response to increased levels of ROS and ROS-mediated signaling activity [58]. The xCT inhibitor, sulfasalazine, was able to ablate this CD44-driven tumor growth [58]. Thus, while CSCs have the ability to mitigate ROS-induced stress through metabolic reprogramming, our ability to precisely control these antioxidant stress response mechanisms may be effective in restricting CSC function.

Until now, very few studies have examined in fine details the manner by which differentiated or non-CSCs can be metabolically reprogrammed to induce dedifferentiation to give rise to CSCs. The landmark stem cell reprogramming study first demonstrated that human fibroblasts could dedifferentiate and form pluripotent stem cells through the overexpression of the “Yamanaka factors”—OCT4, SOX2, KLF4 and c-MYC [59]. NANOG and Lin-28 were also later shown to be important in this “dedifferentiation” or reprogramming process [60,61]. By subjecting glioma, hepatoma, and lung cancer cells to hypoxia, the induced expression of putative cancer stem cell markers (OCT4, NANOG, LIN-28A) and dedifferentiation could be observed [62]. Such hypoxia-induced CSCs were less apoptotic and more resistant to temozolomide, which is used in the treatment of glioblastoma multiforme (GBM) [62]. While the mechanistic underpinnings of metabolic adaption during dedifferentiation events have yet to be clearly elucidated, hypoxia appeared to be pivotal in triggering metabolic changes such as elevated glycolysis, and it would be interesting to clarify how metabolic rewiring could result in a change in cell state from non-CSCs to CSCs.

## 3. Cell State Transitions Are Enabled by Metabolic Plasticity

Changes between the epithelial and mesenchymal states—namely, the epithelial–mesenchymal transition (EMT), and the reverse process, mesenchymal–epithelial transition (MET)—have long been shown to play pivotal roles in cancer pathogenesis. Although the physiological relevance of EMT in vivo remains debated, the phenomenon has been widely observed to mediate metastasis by allowing otherwise epithelial cells to acquire more invasive and motile phenotypes [63,64,65]. Following metastasis, cells revert to their more epithelial state, which facilitates colonization at the distant metastatic site [63,66,67]. Transcriptional regulation of EMT is controlled by the master EMT transcription factors-Snail, Twist1/Twist2, Zeb1/2 and Slug [68]. Cells that have undergone EMT are typically associated with a less differentiated stem-like state that may be more drug resistant [69,70,71]. These cell state transitions are often transient, as the expression of the EMT master regulators are, in turn, responsive to external stimuli and signaling pathways [63,72]. Key signaling pathways and molecular mediators regulating EMT have been very well-characterized. However, the involvement and role of metabolites in EMT is a fledgling research area that is only gaining attention in recent years.

Fumarate hydratase (FH) is an enzyme within the tricarboxylic acid (TCA) cycle that converts fumarate to malate. While mutations and deletions in fumarate hydratase have been previously reported to drive a number of cancer types and are associated with a more aggressive and metastatic phenotype, its mechanistic basis in promoting disease progression was more recently clarified [73,74]. FH loss and consequent fumarate accumulation promoted EMT by inhibiting ten-eleven translocation (TET) demethylation of miR-200ba429 (a cluster of miRNAs known to repress EMT transcription factors Zeb1 and Zeb2) [74,75]. Supplying FH-proficient cells with fumarate was sufficient to recapitulate these EMT-promoting effects. Conversely, supplying FH-deficient cells with alpha-ketoglutarate (alpha-KG) to reactivate alpha-KG-dependent dioxygenases restores expression of miR-200a, and blocked EMT [74]. Similarly, succinate accumulation was reported to epigenetically silence miR200 expression through the inhibition of the demethylating activity of TET enzymes, thereafter inducing EMT in succinate dehydrogenase subunit b (SDHB)-deficient epithelial kidney cells that lack the ability to convert succinate to fumarate [74]. Apart from TET-driven miR200 suppression, loss of SDHB in chromaffin cells led to the epigenetic silencing of keratin-19, a marker associated with the epithelial state [76]. This EMT phenotype in SDHB-deficient cells could be reversed through the use of decitabine, a DNA methylation inhibitor, supporting the role of an epigenetic mechanism. Furthermore, mutations in SDHB have been linked to hypermethylation of promoter CpG islands that promote Snail and Slug activation, pointing to multiple mechanisms through which TCA cycle enzymes could epigenetically regulate the EMT program [76,77].

Isocitrate dehydrogenase (IDH) is another TCA cycle enzyme that converts isocitrate to alpha-ketoglutarate, and IDH1/IDH2 mutations have been reported in a number of cancers including leukemia, melanomas, oligodendrogliomas, and astrocytomas [78]. Mutant IDHs further convert alpha-KG into the oncometabolite 2-hydroxyglutarate, which inhibits the Jumonji-family histone demethylase to increase H3K4 trimethylation within the ZEB1 promoter region to increase its expression, thereby driving EMT [79]. Thus, these studies provided robust observations that the epigenetic control of key cell state-determining genes by distinct metabolites and metabolic pathways is essential for enabling cellular plasticity.

Apart from metabolites which are well-known to control epigenetic processes, alterations in lipid metabolism have been increasingly implicated in EMT. Intriguingly, confounding observations on the role of synthesis and accumulation of fatty acids in EMT could indicate context dependency of these metabolites in specific cancer processes. In some instances, a reduction in fatty acid levels appeared to be important in promoting EMT. Snail1, which mediates TGFβ1-induced EMT, was found to suppress the expression of the key lipogenic transcription regulators, carbohydrate response element binding protein (ChREBP) and sterol regulatory element binding protein (SREBP), in A549 lung adenocarcinoma cells, leading to downregulation of the fatty acid synthase, FASN [80]. FASN knockdown cells gained enhanced expression of mesenchymal markers, were more motile, and also more metastatic in vivo. Predicted Snail1-binding sites within the ChREBP promoter likely led to the direct regulation of ChREBP transcription by Snail1 [80]. Consistent with this, TGFβ1-induced phosphorylation (by TAK1), and thus, inhibition of the fatty acid synthesis enzyme, ACC1 (which catalyzes the conversion of acetyl-CoA to malonyl-CoA), was shown to activate EMT via the accumulation of ACC1’s substrate acetyl-CoA, which led to increased acetylation required for SMAD2 activation [81].

In other instances, however, increased fatty acid synthesis appeared to promote EMT. Stearoyl-CoA desaturase-1 (SCD1), which catalyzed the conversion of stearoyl-CoA and palmitoyl-CoA into the monounsaturated fatty acids oleate and palmitoleate, contributed to β-catenin nuclear localization in MDA-MB-231 breast cancer cells to drive EMT [82]. Apart from breast cancer, SCD1 overexpression was observed in lung adenocarcinoma and colorectal cancer patients, with SCD1 expression levels being correlated with poor prognosis [83,84]. ATP citrate lyase (ACLY), which converts citrate into oxaloacetate and acetyl-CoA, of which the latter may be fed into lipogenesis, has similarly been implicated in EMT. The loss of ACLY was capable of reversing EMT in A549 lung adenocarcinoma cells and promoting a more differentiated cell state [85]. While in hepatocellular carcinoma (HCC), increased fatty acid uptake via upregulated levels of the CD36 transporter activated EMT through Wnt signaling [86]. Treatment of HCC cell lines with palmitate resulted in the activation of Wnt and TGFβ signaling and a more pronounced EMT phenotype that could be abrogated by CD36 inhibition. Taken together, the confounding contributions of fatty acid levels to EMT could point to a delicate balance between energy production and the biosynthesis of precursors required to mediate the morphological changes occurring during EMT [82].

Amino acid metabolism plays crucial roles in cancer cells, fueling the TCA cycle through anaplerosis and supporting macromolecular biosynthesis [87]. Emerging roles for altered amino acid metabolism have also been found in EMT. Using liquid chromatography–mass spectrometry (LC–MS)-based targeted metabolomic analysis, particular amino acids, namely glutamine, glutamate, beta-alanine and glycylleucine, were observed to be enriched across breast cancer cell lines, each overexpressing a unique EMT transcription factor (Snail, Twist and Goosecoid). These amino acids formed an EMT-associated metabolic signature that further demonstrates prognostic value in cancer patient samples [88]. In line with this observation, glutaminase 1 (GLS1), which converts glutamine to glutamate (thereby allowing glutamate to potentially feed back into the TCA cycle through alpha-KG), was observed to be activated by Snail, TGFβ and Wnt pathways to promote EMT, whereas loss of GLS1 inhibited this [89]. The EMT-associated transcription factor, Dlx-2, was found to be responsible for mediating TGFβ- and Wnt signaling-induced GLS1 expression, whereas the perturbation of the glutamine metabolism by GLS1 silencing, glutamine starvation, and the use of inhibitors could arrest EMT (by regulating Snail expression levels), thereby suppressing tumor growth and metastasis [89].

GLS2, the mitochondrial counterpart to GLS1, intriguingly appeared to mediate opposing effects. Breast cancer cells induced into a mesenchymal state through the overexpression of EMT transcription factors were observed to have reduced levels of GLS2, which were associated with less mitochondrial respiration and reduced glutamine dependency under glucose-limiting conditions [90]. Conversely, the inhibition of the EMT transcription factor, FOXC2, in GLS2-depleted cells could restore GLS2 expression and consequent glutamine utilization. Independent of its glutaminase activity, GLS2, surprisingly, was found to stabilize Dicer to promote miR-34a processing and subsequent inhibition of Snail expression in HCC cells to repress the EMT phenotype [91]. Nonetheless, the mechanisms for the metabolism-independent function of this metabolic enzyme remains to be elucidated. The pleiotropic effects of GLS2 could be one account for the discrepancy between the observed effects of GLS1 and GLS2 and warrants further investigation as well. Alternatively, the compartmentalization and subcellular context of glutaminolysis could be the determining factor for its effects on EMT.

Asparagine is an amino acid that has been implicated in several cancer processes. For one, asparagine was found to promote the uptake and metabolism of other amino acids such as serine and arginine in LPS2 liposarcoma cells [92]. In other instances, cancer cells are dependent on asparagine for survival under both glutamine-limiting and glutamine-independent conditions [92,93,94]. Asparagine levels also appeared to be key for the maintenance of the EMT or metastatic state in breast cancer cells. Parallel methods to reduce asparagine bioavailability by limiting asparagine synthesis via knockdown of asparagine synthetase, depleting existing asparagine pools via L-asparaginase treatment, and reducing circulating asparagine levels via dietary restriction, were all effective in limiting the metastatic potential in breast cancer cells without impacting the growth of the primary tumors, thus, pointing to its specificity for the metastasis process [95]. Given well-established evidence suggesting that cancer cells with a mesenchymal phenotype tend to be more aggressive and associated with poorer prognosis, targeting the underlying metabolic pathways that convert cancer cells into an epithelial state may be an attractive therapeutic strategy.

## 4. Metabolic Plasticity Promotes the Acquisition of Drug Resistance

While advances in cancer therapeutics have significantly improved the disease-free survival and quality of life in patients, most treatments ultimately fail when cancer resistance develops. A major cause for drug resistance is the cellular plasticity inherent to cancer cells, enabling them to rapidly change and evolve by adopting different mechanisms. In addition to the well-described signaling, gene regulatory and epigenetic pathways, metabolic reprogramming is now considered as a plausible route by which cancer cells can gain resistance to chemotherapy and tyrosine kinase inhibitors (TKIs).

Cisplatin is one of the chemotherapeutic agents widely used against multiple cancers [96,97]. Although the mechanisms of platinum resistance have been extensively studied, new lines of evidence suggest the involvement of metabolic rewiring [98]. One of the metabolic features of cancer cells is their ability to increase glucose consumption for aerobic glycolysis [7,99]. Increased glycolytic flux was demonstrated to induce cisplatin resistance in several cancer cell lines [100,101,102]. The overexpression of HK2, a rate-limiting enzyme in glycolysis, correlated with resistance in ovarian cancer. This increase in HK2 was shown to enhance cisplatin-induced autophagy through ERK1/2 phosphorylation, while the loss of HK2 in resistant cell lines was able to re-sensitize them to treatment [100]. Consistent with their dependence on glucose metabolism, cisplatin-resistant ovarian and gastric cancer cells were also reported to be sensitive to glucose starvation or inhibition of the glucose metabolism using 2-Deoxy-D-glucose (2-DG) [101,103]. Glycolytic enzyme Enolase 1 (ENO1) was upregulated in cisplatin-resistant cell lines and the loss of ENO1 led to the increased sensitivity to cisplatin [101]. Nevertheless, the connection between cisplatin and its direct mechanistic role in rewiring the cellular metabolism has not been forthcoming.

One of the major demands for increased glycolytic flux is to meet the increased biosynthetic needs of cancer cells. Several intermediates from glycolysis contribute to biosynthetic pathways that generate macromolecules such as nucleic acids, amino acids or lipids [10,99]. The pentose phosphate pathway (PPP), which runs parallel to glycolysis, protects cancer cells against oxidative stress by providing NADPH [102,104]. In addition, the increased expression of the PPP’s rate-limiting enzyme, glucose-6-phosphate dehydrogenase (G6PDH), as well as its downstream metabolite, glutathione (GSH), were reported in cisplatin-resistant lung and ovarian cell lines [105,106,107]. These resistant cancer cells demonstrated an increased glutamine/glutamate flux, mediated by cystine/glutamate antiporter xCT, which aided in glutathione synthesis to combat oxidative stress [108,109,110]. Using G6PDH inhibitors, 6-amino nicotinamide (6-AN) and dehydroepiandrosterone (DHEA), re-sensitization to cisplatin could be achieved [105,106]. These metabolic changes demonstrate an enhanced dependence on glucose metabolism in response to cisplatin resistance.

Apart from glucose dependence, an altered amino acid metabolism represents another adaptation to platinum-based drug resistance [111,112]. Amino acids such as glutamine and tryptophan were found to be preferentially utilized over glucose in resistant lung cancer cells through the activation of the kynurenine pathway. In addition, indoleamine 2,3-dioxygenase-1 (IDO1), the rate-limiting enzyme which converts tryptophan to kynurenines, was upregulated in these cells. Increased resistance to cisplatin correlated with increased sensitivity to IDO1 inhibitor presented a therapeutic window for overcoming cancer resistance [113]. Increased dependence on glutamine metabolism through the upregulation of glutamine transporters ASCT2 and GLS was also observed in cisplatin-resistant ovarian cancer cells, which were more sensitive to glutamine deprivation by GLS knockdown as compared to their cisplatin-sensitive counterparts [114,115]. Encouragingly, combination treatment with glutaminase inhibitor, BPTES, showed synergistic effects in overcoming cisplatin resistance [115,116,117].

Alterations in lipid metabolism have also been implicated in chemotherapy-induced drug resistance. Carnitine palmitoyltransferase-2 (CPT2), a rate-limiting enzyme of FAO, was downregulated in cisplatin-resistant hepatocellular carcinoma. The silencing of CPT2 contributed towards resistance through increased lipogenesis, through the upregulated expression of the lipogenic enzyme, SCD1, although the mechanistic underpinnings remained unclear [118]. On the contrary, the activation of FAO, driven by the JAK/STAT3 signaling pathway through regulation of CPT1B (a key enzyme facilitating FAO), was seen in paclitaxel-resistant breast cancer [48]. Mirroring observations in EMT, the seemingly confounding roles of lipid metabolism as an important metabolic pathway for conferring chemotherapy drug resistance require further investigations.

Metabolic changes have also been reported in resistance against targeted therapies such as tyrosine kinase inhibitors (TKIs) [119]. In NSCLCs, EGFR TKIs that include gefitinib are commonly used to treat mutant EGFR-driven tumors, prevalent in Asian never-smokers [120]. Gefitinib-resistant cells appeared able to switch their metabolic reliance from glucose consumption to oxidative phosphorylation by increasing mitochondria activity. It was observed that these cells expressed high levels of MCT-1, which indirectly resulted in enhanced OxPhos, with the upregulation of lactate dehydrogenase B (LDHB). The inhibition of the MCT-1 transporter with the small molecule inhibitor, AZD3965, was able to decrease cell viability, migratory abilities and mitochondrial bioenergetics of cancer cells in serum-limiting conditions [121]. Similarly, in HER2-driven breast cancer, HER2-targeting TKIs such as lapatinib are often used to treat advanced breast cancer [122,123]. While lapatinib reduced the expression of estrogen-related receptor alpha (ERRα), a nuclear receptor that regulates mitochondria biogenesis, lapatinib-resistant cells were able to re-express ERRα through activated mTOR signaling. This re-expression of ERRα provide the necessary metabolic adaptation through enhancing the glutamine metabolism. Inhibiting ERRα was shown to reverse this metabolic rewiring and re-sensitized cells to lapatinib treatment [124]. BRAF mutations, which are often present in more than half of melanoma patients, are treated with selective MAPK inhibitors. However, initial tumor regression is often followed by the acquisition of drug resistance and cancer relapse [125,126]. In BRAF inhibitor-resistant melanoma, high dependence on mitochondria for survival, with increased OxPhos and mitochondrial biogenesis were observed [127,128]. These examples highlight the manner by which alterations to mitochondrial activity could have a role in supporting targeted therapy resistance.

Alterations to the branched-chain amino acid (BCAA) metabolism represents yet another path towards gefitinib resistance [129]. BCAT1, a cytosolic aminotransferase that catalyzes the catabolism of BCAA, was reported to be epigenetically upregulated by H3K9 demethylation in NSCLC. Since gefitinib treatment promoted ROS accumulation in gefitinib-resistant cells, BCAT1 was observed to scavenge ROS through GSH synthesis. Consequently, the combination of gefitinib with ROS-inducing agents such as piperlongumine or phenethyl isothiocyanate, or the inhibition of GSH synthesis was able to overcome TKI resistance [129,130]. Likewise, in BRAF inhibitor-resistant melanoma, tumor cells were more reliant on glutamine as an alternate carbon source. Only BRAF inhibitor-resistant cells demonstrated sensitivity to a GLS inhibitor, highlighting a potential strategy for overcoming BRAF inhibitor resistance [127,128]. Nonetheless, the precise manner by which TKI resistance is directly linked to amino acid metabolism remains to be elucidated.

While acquired resistance represents a major adaptive force of cancer cells, intrinsic resistance to TKI is not uncommon. Owing to tumor heterogeneity, in melanoma, a subset of mutant BRAF cells was found to be intrinsically resistant to MAPK inhibitors. At the basal level, these BRAF inhibitor-resistant cancer cells maintain low levels of mitochondria mass and biogenesis, regulated by TFAM and TRAP1 to overcome the MAPK inhibitor treatment. Treatment with the MAPK inhibitor further induced the activation of OxPhos through mechanisms which are yet unknown. By targeting mitochondrial biogenesis using a HSP90 inhibitor, mitochondrial dysfunction was induced, and tumor growth was abrogated [128]. These examples, therefore, underscore the important roles for metabolic adaptability in enabling both acquired and intrinsic TKI resistance.

Modifications to the tumor microenvironment (TME) as a result of tumor- or stroma-secreted metabolites have, more recently, been found to contribute towards therapy resistance [48,129,131,132]. In NSCLC cancer cells resistant to EGFR or MET inhibition, a metabolic shift towards increased glycolysis and lactate production resulted in acidification of the TME. The stimulation of surrounding cancer-associated fibroblasts (CAFs) increased production of HGF-sustained TKI resistance through the activation of MET-dependent signaling [131]. In a similar vein, metabolites secreted into the TME also facilitate therapy resistance. Chemo-protective lipid mediators such as leptin, secreted by surrounding adipocytes, enhanced the STAT3 activated-FAO pathway, contributing to paclitaxel resistance and CSC self-renewal in breast cancer [48,129]. Arachidonic acid (AA), a polyunsaturated fatty acid, also secreted by adipocytes in ovarian cancer, was reported to enhance cisplatin resistance. AA was thought to directly activate the Akt signaling pathway, thereby inhibiting apoptosis [129].

Given the pleiotropic ways that altered metabolism can contribute to chemotherapy and TKI resistance, it is unsurprising that many combination treatments with novel metabolic drugs are now underway. However, very few such drugs or combinatorial treatments have progressed into advanced clinical trial phases. This highlights the greater imperative to understand the complex metabolic phenotypes of heterogenous tumors and how one might more precisely apply such metabolic drugs either singly or in combination with standard-of-care therapies.

## 5. Metabolic Interventions for Restricting Cancer Progression

The extensive metabolic rewiring that is imposed during cell state transitions raises the possibility of deploying new metabolic inhibitors as interventions at specific contexts in disease progression. A myriad of compounds that target metabolic pathways in cancers have been, or are being, tested in clinical trials (Table 1). A major caveat is potential toxicity issues, as many of the metabolic pathways are also central to normal cell functions. Therefore, it is necessary to identify viable therapeutic windows that only disrupt metabolic dependencies in tumors, while sparing normal tissues and organs.

As increased glycolysis and enhanced glucose uptake can support acquired resistance to chemotherapies and TKIs, several inhibitors targeting these processes have been developed. Key enzymes such as HKs, LDHA and G6DPH, are often overexpressed in cancers and serve as possible therapeutic targets. Since CSCs also appear to rely more on glycolysis, attempts to selectively target CSCs were undertaken with glycolysis inhibitors such as 2-DG. 2-DG appeared to impact CSC populations with mitochondrial defects in triple-negative breast cancer (TNBC) [151]. In addition, the efficacy of 2-DG was demonstrated against several cancer types in pre-clinical studies using mouse xenograft models and human cell lines [152,153,154]. However, several other studies have found that the administration of high levels of 2DG to patients reduced tumor burden but also led to hypoglycemia symptoms. Lower doses, while mitigating side effects, were met with compromised efficacy [155]. Likewise, while the compounds 6-AN and DHEA could effectively inhibit G6PD [105,106,156], none has been met with clinical success. Administration with 6-AN revealed toxicity and neurological disturbance at high dosage, whereas low dosage failed to demonstrate efficacy [157]. DHEA, which is an endogenous precursor hormone produced by the adrenal glands, was also unsuccessful during pre-clinical development due to the high oral dosage required, as well as its rapid conversion into active steroids by the body [158,159]. Efforts were later concentrated on developing LDHA inhibitors, but none have entered clinical trials after being plagued with issues of toxicity, poor drug bioavailability, and the lack of LDHA dependence in patient tumors [160]. These studies unequivocally highlighted the insurmountable challenges of drugging glucose metabolism pathways in cancer.

Disrupting mitochondrial function has been considered an avenue for restricting the ability of CSCs to switch to OxPhos for deriving energy. A tool compound, XCT790, which inhibits the ERRα-PGC1 signaling pathway, was deployed to disrupt mitochondrial biogenesis; it was effective in reducing OxPhos in tumor-initiating stem-like cells in breast cancers [161]. In addition, a lead compound, C29, that targets ERRα, was effective in sensitizing HER2+ lapatinib resistance cells to lapatinib treatment [124]. Furthermore, IACS-010759, an inhibitor of mitochondrial complex I, is currently undergoing Phase I clinical trials, having demonstrated a strong preclinical efficacy in inhibiting acute myeloid leukemia (AML) and brain cancer that were shown to be reliant on OxPhos [162].

L-asparaginase, a bacterial-derived metabolic enzyme responsible for converting glutamine to glutamate, was previously identified to be promising in treating acute lymphoblastic leukemia (ALL). However, this was undermined by adverse side effects, such as immune–allergic responses and hepatotoxicities [163,164]. A recent technological advancement has allowed for L-asparaginase to be loaded onto red blood cells (ERY001), thereby improving its half-life and drastically reducing related toxicities and other adverse side-effects [165]. ERY001, in combination with chemotherapy, is currently undergoing Phase III clinical trials for use as second-line therapy against advanced pancreatic adenocarcinoma [138]. To address the issue of targeting specific cell states, it was found that depriving luminal breast cancer cells of glutamine via the glutamine analog 6-diazo-5-oxo-L-norleucine (DON) or two GLS1 inhibitors (compound 968 and BPTES) could suppress TGF-β-induced EMT [89]. BPTES appeared to re-sensitize otherwise resistant melanoma cells to BRAF inhibition, as well as cisplatin-resistant ovarian and breast cancer cells to chemotherapy, owing to their reliance on the glutamine metabolism [115,116,117,127]. Targeting the glutamine metabolism may be useful for limiting EMT-driven metastasis, as well as reversing resistance, and clinical trials involving an alternative GLS1 inhibitor, CB-839, are underway [166].

As highlighted previously, FAO has been shown to be reprogrammed in both tumor and its microenvironmental cells [150,167]. Etomoxir, a CPT1 inhibitor that blocks FAO, was preferentially effective in targeting basal-like triple-negative breast cancer cells with high MYC expression [168]. Perhexiline, another CPT1 inhibitor, disrupted FAO in paclitaxel-resistant breast cancer cells and re-sensitized them to treatment [48]. Unfortunately, there are presently no ongoing clinical trials relating to CPT1 inhibition in the context of cancer, thereby presenting an unmet clinical need for overcoming FAO dependency. The utilization of FAO by cancer cells is tightly connected to the availability of fatty acids. Increased fatty acid uptake mediated by upregulated CD36 transporter levels was observed to promote metastasis in gastric cancer, oral squamous cell carcinoma, ovarian cancer, as well as HCC [86,169,170,171]. A CD36 chemical inhibitor, sulfo-N-succinimidyl oleate (SSO), which blocks the fatty acid-binding pocket on CD36, could reduce cell migration and mitigate an EMT phenotype triggered by the exposure of HCC cell lines to free fatty acid [86,172]. Neutralizing antibodies against CD36 were also shown to inhibit metastasis in both immunodeficient and immunocompetent orthotopic mouse models of human oral cancer [170]. Although these direct methods of CD36 inhibition have not progressed to clinical trials, the use of thrombospondin-1 (TSP-1) mimetics, such as ABT-510 and CVX-045, which inhibit CD36-mediated fatty acid uptake, were explored [136,173]. However, these trials were terminated due to severe side effects and lack of efficacy [136]. Drugging FAO and fatty acid uptake thus remain a formidable obstacle that may require improved drug delivery and targeting mechanisms that mitigate issues of toxicity.

Efforts to drug the methionine pathway in cancer have been making some headway. To exploit the dependency on exogenous methionine by CSCs and other methionine-dependent cancers, AG-270, an allosteric MAT2A inhibitor, was developed and is currently undergoing Phase I clinical trials. Early results indicated its tolerability in patients and its anti-tumoral effects when applied in combination with taxanes and gemcitabine [174]. Similarly, targeting MCT-1 was demonstrated to be effective in reducing the stemness of CSCs, as well as abrogating gefitinib-resistant cells [46,121]. The first-in-human first-in-class trial of the MCT-1 inhibitor, AZD3965, is currently undergoing a Phase I clinical trial for Burkitt lymphoma, diffuse large B cell lymphoma and advanced solid tumors [175]. By blocking the lactate export in highly glycolytic cancer cells or inhibiting the lactate uptake in lactate-dependent cancers, AZD3965 has displayed encouraging preclinical and early Phase I study results [176].

Given that the accumulation of 2-HG from mutant IDH1/IDH2 is a key inducer of EMT, and that knockdown of IDH1 in mutant IDH1-overexpressing HCT116 colorectal cancer cells could reverse the EMT phenotype, targeting mutant IDH to prevent 2-HG production has been considered [177]. Notably, several small molecule inhibitors with exquisite specificity for mutant IDH1 and IDH2 have been developed, and these do not impact the function of the wildtype proteins [178]. Whereas Ivosidenib (AG-120) and Enasidenib (AG-221) are FDA-approved for the treatment of relapsed or refractory IDH-mutant AML, trials are ongoing to assess the effectiveness of various IDH inhibitors in the context of solid tumors such as gliomas [145]. Because 2-HG accumulation was shown to result in DNA and histone hypermethylation, there are also a number of trials exploring the combination of IDH inhibitors with epigenetic drugs such as azacytidine [179]. Although IDH inhibitors have not been used explicitly to show the reversion of EMT, their efficacy against various cancers hold promise [145].

**Figure 1:**
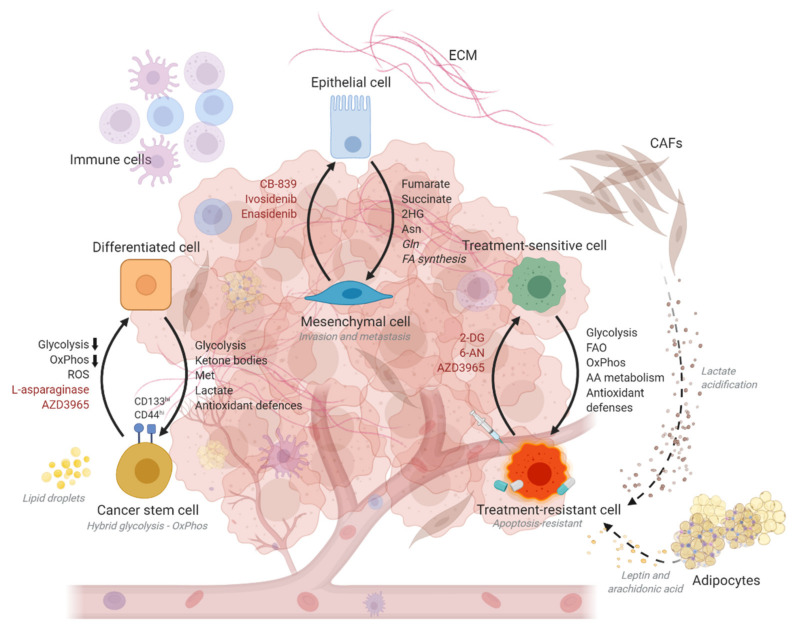
Schematic diagram illustrating the metabolic alterations underlying the shifts between differentiated versus cancer stem cell states; epithelial and mesenchymal states; and treatment-sensitive or resistant states. Crucial interactions with the tumor microenvironment that are key determinants of metabolic rewiring within the cancer cells are also depicted. Upregulated metabolites and processes are indicated in black. Downregulated metabolic processes are indicated with a black downward-pointing arrow. Processes for which evidence remains confounding are italicized. Metabolic drugs capable of causing a shift in cell states are indicated in red. ECM, extracellular matrix; CAFs, cancer-associated fibroblasts; Asn, asparagine; Gln, glutamine; AA, amino acid; OxPhos, oxidative phosphorylation; ROS, reactive oxygen species; Met, methionine; 2HG, 2-hydroxyglutarate; FA, fatty acid; FAO, fatty acid oxidation.

Metabolic adaptations drive cellular plasticity as cancer cells need to rewire their cellular metabolism to adapt to alternate cell states during disease progression. An interplay of distinct metabolic changes collectively underlies the shift between a stem-like versus a more differentiated state, an epithelial versus a mesenchymal phenotype, and a resistance versus a treatment-naïve cell state (Figure 1).

## 6. Conclusions

Changes in the metabolic preferences of cancer cells are responsible for mediating phenotypic changes through a combination of altered signaling pathways, transcriptional regulation, as well as epigenetic control. With the paradigm shift towards precision oncology, our understanding of cancer metabolism is likely to be integrated with current genomic, molecular and histological assays used to aid clinicians in disease treatment stratification, real-time tumor tracking, and in tailoring customized therapy. The challenge for researchers will be to address the complexity underlying these metabolic vulnerabilities in order to discover new therapeutic strategies that effectively abrogate the contribution of cellular plasticity to tumor initiation and progression.

## Figures and Tables

**Table 1:** Summary of metabolic drug targets and related clinical trials. AML: acute myeloid leukemia; IDH: isocitrate dehydrogenase.

Compound	Target(s)	Reason for Termination/Clinical Trial No.	Phase	Indications	Ref
**Discontinued**					
2DG	HK1	Short half-life Hypoglycemia NCT00633087	I	Prostate cancer	[133,134]
Etomoxir	Carnitine palmitoyl-transferase	Completed	I/II	Congestive heart failure	[135]
ABT-510	CD36	Adverse side effects and lack of efficacy NCT00073125 NCT00061672 NCT00061659	II	Renal cell carcinoma, Non-Hodgkin lymphoma, Hodgkin’s lymphoma, Soft tissue sarcoma,	[136]
CVX-045	CD36	Adverse side effects and lack of efficacy NCT00879554	I	Advanced solid tumors, neoplasms and carcinoma	[136]
**In clinical trials**					
IACS-010759	Mitochondrial Complex I	NCT03291938	I	Advanced cancers	[137]
ERY001	Glutaminase	NCT02195180	III	Progressive metastatic pancreatic carcinoma	[138]
CB-839	Glutaminase	NCT03965845	Ib/II	Combination therapy with CDK4/6i Palbociclib in advanced/metastatic solid tumors.	[139]
AG-270	MAT2A	NCT03435250	I	Advanced solid tumors or lymphoma with methylthioadenosine phosphorylase (MTAP) loss	[140]
AZD3965	MCT1	NCT01791595	I	Adult solid tumor Diffuse Large B Cell Lymphoma Burkitt Lymphoma	[141,142]
Ivosidenib (AG-120)	IDH1	NCT02073994	I/II	FDA-approved for adult relapsed or refractory AML with IDH1 mutations In trials for glioma, cholangiocarcinoma, cholangiosarcoma	[143,144]
BAY-1436032	IDH1	NCT03127735, NCT02746081	I	AML and solid tumors (including glioma)	[145]
IDH305	IDH1	NCT02381886	I	IDH-mutant glioma, AML/Myelodysplastic syndromes (MDS), and other solid tumors	[146]
FT-2102	IDH1	NCT02719574	I/II	Monotherapy and as combination therapy with azacitidine for AML and MDS	[145]
Enasidenib (AG-221)	IDH2	NCT01915498, NCT02273739	I/II	FDA-approved for relapsed or refractory AML In trials for advanced hematologic malignancies with IDH2 mutation, solid tumors, glioma, intrahepatic cholangiocarcinoma	[147,148]
Vorasidenib (AG-881)	IDH1/IDH2	NCT02492737, NCT02481154	I/II	Advanced hematologic malignancies with IDH1/IDH2 mutation, glioma, cholangiocarcinoma, cholangiosarcoma	[149,150]
